# A New CPW-Fed Semicircular Inverted Triangular Shaped Antenna Based on Mixed-Alternate Approach for 5G Millimeter-Wave Wireless Applications

**DOI:** 10.3390/mi14010220

**Published:** 2023-01-15

**Authors:** Permanand Soothar, Hao Wang, Zaheer Ahmed Dayo, Yu Quan

**Affiliations:** 1 School of Electronic and Optical Engineering, Nanjing University of Science and Technology, Nanjing 210094, China; 2 Department of Electronic and Telecommunication Engineering, Mehran University of Engineering & Technology, Jamshoro 76062, Pakistan; 3 College of Computer Science, Huanggang Normal University, Huangzhou 438000, China

**Keywords:** alternate-mixed approach, CPW-fed, broadband, high gain, partial defected metal ground plane, semicircular inverted triangular-shaped, 5G millimeter-wave

## Abstract

This paper presents the design and development of a new semicircular inverted triangular shaped antenna for 5G millimeter-wave wireless applications. An alternate-mixed approach based on cavity, slots and loaded stubs is employed in the designed antenna lattice. The suggested antenna structure is formed by a radiator, partial defected metal ground plane and a 50 Ω coplanar waveguide. The proposed antenna resonated at multiple frequencies by the setting up of the proper dimensions and locations of the rectangles, elliptical cut slots and cavity stubs. Furthermore, a parametric analysis is carried out to examine the antenna’s effectiveness and impedance-matching controls. The proposed structure is realized on the low-cost RT/Duroid Rogers RO3010™ laminate with an overall small size of 1.381λ_0_ × 1.08λ_0_ × 0.098λ_0_, where λ_0_ represents the wavelength corresponding to the minimum edge frequency of the 23 GHz at 10 dB impedance bandwidth of the antenna. The antenna’s key characteristics in terms of bandwidth, gain, radiation patterns and current distribution have been investigated. The antenna exhibits high performance, including an impedance bandwidth of 19 GHz ranging from 23 GHz to 42 GHz, results in 58.46% wider relative bandwidth calculated at 10 dB scaled return loss, a peak realized gain of 6.75 dBi, optimal radiation efficiency of 89%, stable omnidirectional-shaped radiation patterns and robust current distribution across the antenna structure at multiple resonances. The designed antenna has been fabricated and simulation experiments evaluated its performance. The results demonstrate that the antenna is appropriate and can be well integrated into 5G millimeter-wave wireless communication systems.

## 1. Introduction

Recently, massive development in 5G networks have been at the forefront of research. The millimeter-Wave (mm-Wave) communication spectrum has received much attention. There has been a great deal of interest focused on effective antenna designs which offer higher gains and broader bandwidth for use in a future 5G mm-Wave system [1]. High frequencies in the mm-Wave range are the only examples with this capability, hence upgraded spectrum utilization will be necessary to cover the specific range of frequencies [2]. A spectrum utilization at frequencies greater than 24 GHz has been suggested by the World Radio Communication (WRC) conference and recommendations sent to International Telecommunication Union Radio (ITU-R) to nominate potential frequency bands [2]. UP to this point, frequency bands between 24.25 and 86 GHz have been reported in the literature [3]. However, 28 GHz and 38 GHz frequency bands are the best choice for modern 5G communication technologies [4].

There are different design methods available for upcoming 5G-related systems, particularly for a high frequency spectrum [5,6]. Furthermore, numerous design techniques have achieved the high performance features necessary to meet the requirements of 5G systems, including broad bandwidth, high gain, narrow beam width and small size [7,8,9]. In the literature, special antenna structures have been designed particularly to address 5G wireless system requirements, such as substrate integrated waveguide (SIW-fed), multi-layered SIW cavity-fed and coplanar waveguide (CPW-fed) [10,11,12]. However, SIW technology exhibits low transmission loss characteristics and the reported antenna designs require different laminate layers, bonding films and a CPW to SIW wall, which makes them complex and challenging to assemble. CPW-fed partially defected metal ground plane (PDMGP) antennae are simple to design and easy to fabricate and integrate into modern wireless systems. Moreover, the CPW-fed PDMGP planar antenna is a good choice, with an easy fabrication process, cost-effectiveness and good performance features. Hence, it is important to achieve an antenna that radiates with a broad impedance bandwidth and high gain in order to reduce the effects of geometrical complexity, which are more noticeable in the 5G mm-Wave spectrum [13,14].

In the past five years, researchers have proposed various strategies and have modified slot antenna designs to attain broad impedance bandwidth and high gain features. These approaches have mainly focused on different feeding networks [15,16,17,18,19,20,21], defected ground structures (DGS) [22,23,24], artificial magnetic conductors (AMC) loaded lattice [25] and the etching of cut slits from radiators [26,27,28,29]. However, few of these compact and low profile antenna design structures were capable of covering a broad bandwidth at 5G mm-Wave frequency range. A directive broadband Vivaldi antenna employing SIW technology has been investigated in [30,31,32]. The reported antennae covered bandwidth (BW) from 26–40 GHz with a good gain at Ka-band. The developed antennae attained 42.4% and 50% BW but require complicated assembly and high fabrication processes. Besides, reconfigurable H-shaped slot loaded antennae for 28/38 GHz for mobile communications have been suggested [33]. The reported antennae have complex geometries and operate at a certain frequency span. The authors designed microstrip patches and a waveguide aperture antenna with embedded slots to obtain optimal performance, with an increased number of radiating elements at the 28 GHz frequency band [34]. Another bowtie-shaped antenna was designed on Rogers RT/Duroid 5880 substrate embedded with long wire supported by DGS to cover a relative BW of 27–29 GHz and with large dimensions [35]. CPW-fed wideband reflector corner bent antenna elements have been proposed for 5G terminals [36]. The authors constructed the antenna on Nelco NY9220 substrate and achieved BW from 27–33 GHz with 7.2 dBi gain. A compact CPW-fed wideband rectangular ring patch monopole antenna over a square AMC reflector surface with vertical ground stub was investigated in [37]. The reported antenna structure has computational complexity but obtained the impedance bandwidth needed for mm-Wave applications. 

Furthermore, an inverted L-shaped with partial ground MIMO antenna has been investigated in [38]. The antenna achieved a broad BW ranging from 26–40 GHz with larger substrate thickness. Moreover, a new compact single input single output (SISO) slotted-decagon patch antenna with two rectangular grooves at the end of the radiator were presented in [39]. The eight-pointed star-shaped decahedral antenna covered a BW from 23.1–29.94 GHz with an average gain of 6.5 dBi. The authors demonstrated a new printed planar elliptical patch loaded shape for the mm-Wave spectrum [40]. The antenna exhibited a BW of 26.4–31.6 GHz and a peak gain of 8.0 dBi with good broadside radiation characteristics. Another work on a microstrip patch antenna with a slotted ground plane covering the frequency range 26.154–31 GHz, a gain of 5.22 dBi and larger dimensions for 5G communications is outlined in [41]. Several design elements based on different slot shapes have been suggested to achieve an acceptable desired BW [42,43]. However, the design structures were complex, with high manufacturing precision and narrower BW. A multiband Y-shaped antenna for IoT enabled with 5G communications is presented [44]. The designed antenna was embedded on laminate and two equal-length arms were connected with the central part of the flame structure to achieve multiple resonances at the desired band of interest. In addition, a broadband symmetrical E-shaped patch with a multimode resonance antenna has been presented in [45]. The reported antenna consists of an intricate structure with a broad BW ranging from 31.5–50 GHz and exhibited satisfactory performance. However, the work reported high fabrication processes and complicated assembly. Hence, it is concluded from the recently published research that the reported antennae have complex geometries, high fabrication processes, larger dimensions, and most provided reasonable performance across a short frequency range. Furthermore, it is also noted that there is trade-off between antenna key features and compact dimensions. 

The main contributions of the paper are given as follows:

- An alternate-mixed approach based on cavity, slots and loaded stubs is proposed which results in reducing the antenna’s design and fabrication complexity.
- The suggested antenna structure validates a new semicircular inverted triangular-shaped cavity-based antenna with a small size of 1.381λ_0_ × 1.08λ_0_ × 0.098λ_0_, which develops a more effective model.
- The antenna exhibits far-field stable omnidirectional-shaped radiation pattern, broad fractional BW of 58.46% at required frequencies, a peak realized gain of 6.75 dBi and optimal radiation efficiency better than 89% over the operating band. To the author’s best knowledge, the results are more effective than the rest of the previously researched antenna designs.
- Finally, the antenna performance is evaluated and analyzed alongside recently published state-of-the-art works.

The paper suggests a new antenna design to achieve broadband performance for 5G mm-Wave wireless applications. A semicircular inverted triangular shaped antenna is formed with a radiator, PDMGP and a 50 Ω CPW. An alternate-mixed approach is employed to achieve optimal results. The proposed antenna structure is designed on a low-cost Rogers RT/Duroid 3010^TM^ laminate. The antenna exhibited 19 GHz bandwidth (BW) resulting in 58.46% relative impedance BW at 10 dB return loss. Moreover, the antenna obtained high-performance characteristics, including high realized gain 6.75 dBi, strong surface current distribution and stable far-field elevation (E) and azimuth (H) plane patterns. Moreover, an elliptical cavity-based stub loaded element influences the far-field radiation pattern. A high-frequency structure simulator (HFSS 17.2) full wave electromagnetic solver based on the finite element method (FEM) was used in the designing and simulation process. The antenna results have been verified in a real time environment. The simulation and measurement results coincide well each other. The proposed antenna is highly suitable for cutting-edge technology applications and may be integrated into modern 5G mm-Wave communication systems. 

The remainder of the article is organized as follows. The indicated design elements, antenna configuration and proposed antenna model strategy are presented in Section 2. Section 3 includes illustrations of surface current distribution and the effects of various variables. A study of the simulated and measured findings for the suggested antenna is presented in Section 4. A comparison with recently released state-of-the-art work is described in Section 5. Finally, the conclusion is given in Section 6. 

## 2. Proposed Methodology

### Principle of Antenna Operation Mechanism

**Figure 1:**
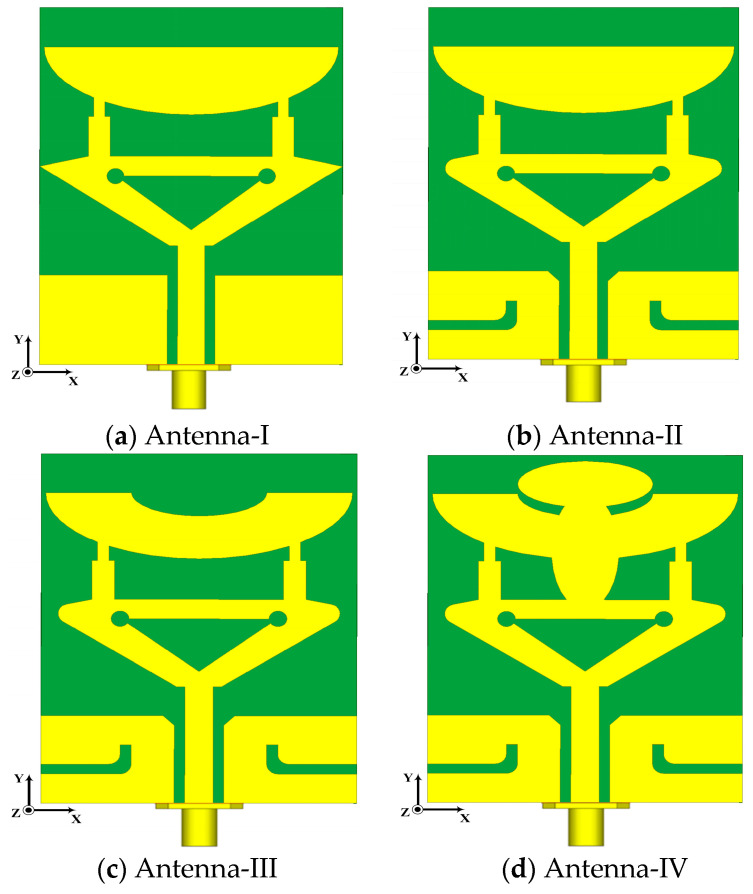

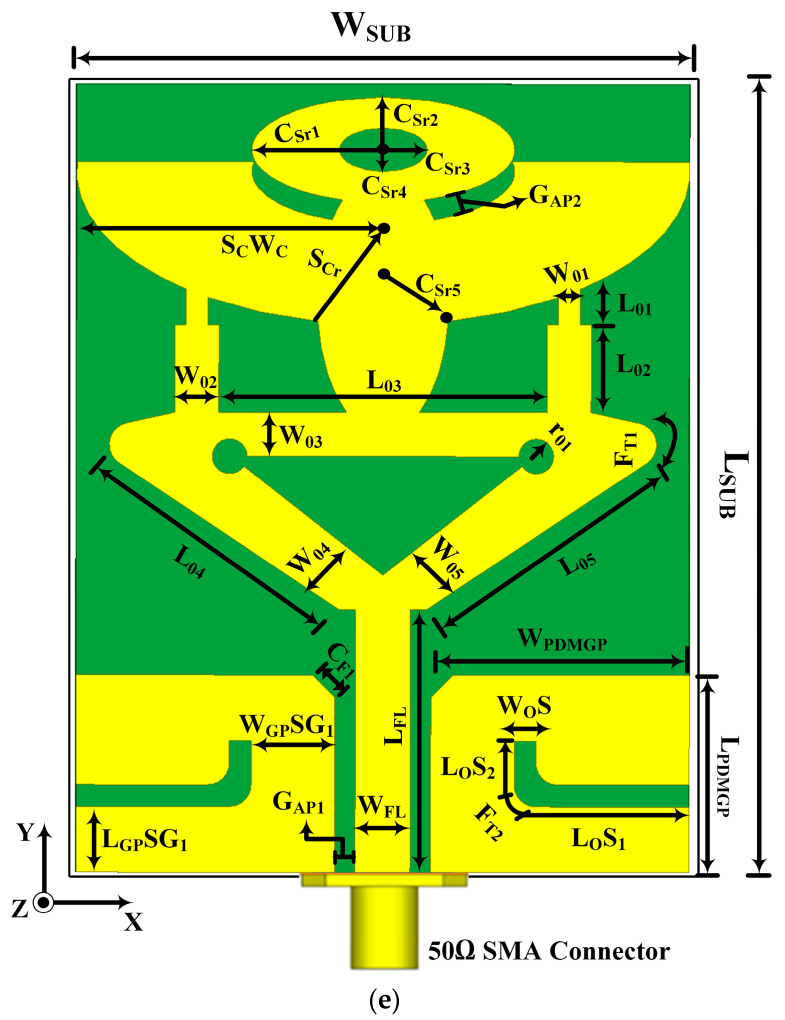
Development stages of proposed antenna:(**a**) simple S_C_IT_S_ CPW-fed with partial defected metal ground plane (PDMGP), (**b**) loaded L-stub with PDMGP and fillet radiator, (**c**) truncated semicircular (S_C_) radiator (**d**) loaded elliptically cavity-based (C_B_) stubs, (**e**) proposed antenna design geometries (top view).

This section explains the proposed antenna mechanism operation, development stages and employed methodology. The antenna development phases are depicted in Figure 1a–d. The model employs an alternate-mixed approach and the overall antenna structure comprises an inverted triangular-shape, rectangle arms, elliptical cavity stubs, dielectric substrate, conducting material sheet (CMS), partial defected metal ground plane (PDMGP) and 50 Ω CPW feeding structure. These antenna components are etched on top of the low-cost Roger RT/Duroid 3010^TM^ printed circuit board (PCB) laminate material with constant dielectric of relative permittivity $\varepsilon_{r}$ = 11.2 and dielectric loss tangent δ = 0.0022. The front and lateral views of the radiator are illustrated in Figure 1e. The yellow and green regions represent the metallic radiating patch and the dielectric laminate. The design of semicircular inverted triangular-shaped (S_C_IT_S_) slots and PDMGP with CPW feedline above the substrate forms the foundation of the envisaged radiator procedure. The CMS is placed on top of the substrate with a standard thickness of 0.035 mm. The antenna is positioned in an x-y position that is parallel to the *z*-axis. 

**Figure 2:**
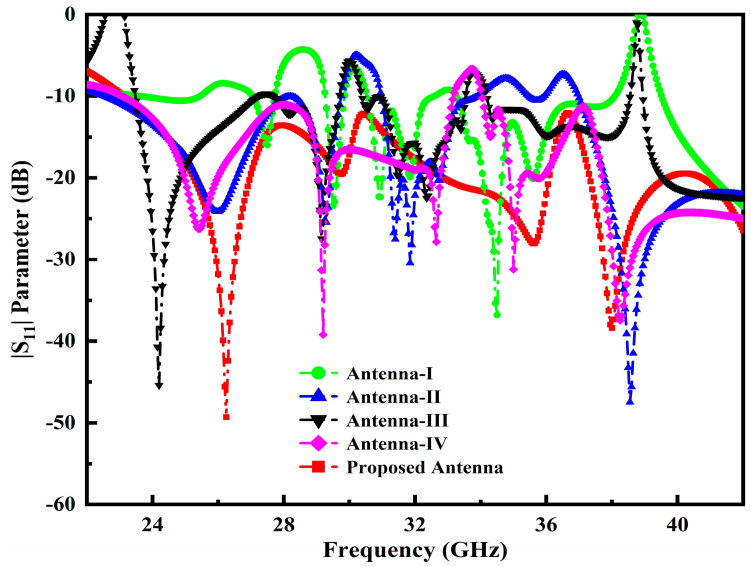
|S_11_| performance across the operable frequency span of the developed antenna prototypes.

Generally, it is challenging to attain perfect matching, particularly at a higher frequency band spectrum, for 5G mm-Wave wireless communication systems. In order to address this problem, further modification has been made to the initial designed antenna dimensions by loading the L-stub slot with PDMGP to obtain impedance BW, high gain and perfect matching, as illustrated in Figure 1b. Moreover, the symmetrically L-loaded slits are truncated by filleting the upper and lower edge corners (F_T2_) on both sides of the PDMGP. The gap of 0.475 mm between the CPW feeding network and PDMGP structures is carefully optimized to achieve 50 Ω matching. The simulated result of the return loss |S_11_| parameter shows perfect matching (blue curve) as depicted in Figure 2. However, the main purpose of a design antenna is to achieve broad BW and high gain. To improve impedance matching, a further modified structure is used with an elliptically cut slit in a semicircular shape (S_C_), as shown in Figure 1c. Fillet (F_T1_) operation is set at the inverted triangular-shaped (IT_S_) corner edges and chamfering (C_F1_) cut into PDMGP on both sides. After performing a truncated S_C_-shaped elliptical stub, significant matching is obtained. To arrive at the desired objective, two more elliptical cavity radial stubs (C_Sr1_ and C_Sr5_) are interconnected with the antenna, as depicted in Figure 1d. To achieve broad BW optimal results, we have engraved another elliptical cavity slot stub, C_Sr3_, cut from C_Sr1_. Moreover, a symmetrical loaded L-shaped slot and an IT_S_ with cavity radial stubs form the finalized compact radiating patch antenna prototype, as displayed in Figure 1e. The proposed antenna possesses overall small dimensions of 1.381λ_0_ × 1.08λ_0_ × 0.098λ_0_. 

Table 1 constructs the specification of the proposed antenna’s optimal variables. To achieve appropriate impedance matching, it is essential to consider the PDMGP’s width, length, radial cavity stubs, inverted triangle shape and substrate.

**Figure 3:**
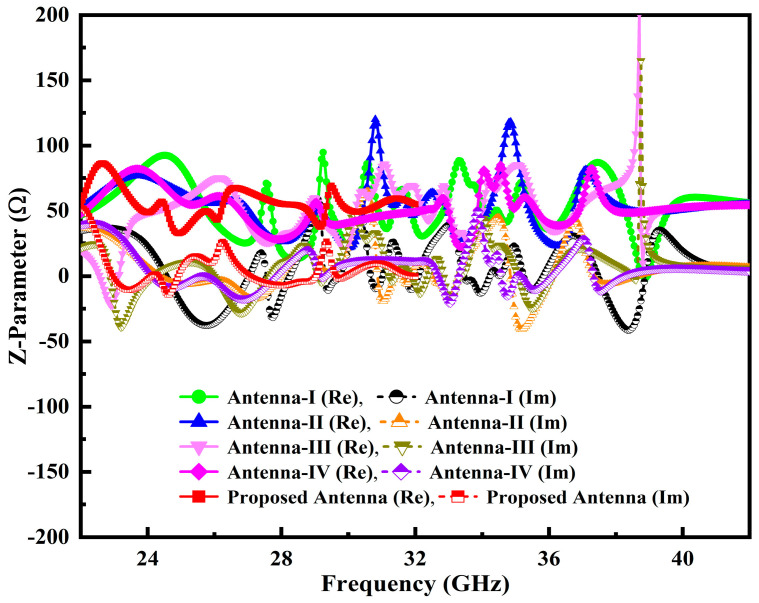
Z-parameter performance across the operable frequency span of the developed antenna prototypes.

Additionally, the antenna performance is significantly impacted by the inserting slots and their locations, tuning stubs and the gap (G_AP1_) between the PDMGP and CPW-feed; as a result, a broad impedance BW, enhanced high gain and flawless matching over a broad frequency range are achieved. The return loss |S_11_| parameter performance of the developed antenna prototypes is elucidated in Figure 2. It can be demonstrated that the proposed antenna structure obtained the perfect matching of fractional BW (red curve) from 23–42 GHz. Figure 3 depicts the simulated variation of the real and imaginary parts of the z-parameter. The plot shows that the input impedance of the port of the developed antenna was matched with the normalized z value of 50 Ω at the operable resonances. 

## 3. Simulation Results and Analysis

This section describes the analysis of the parametric study used to develop the proposed antenna design. The variables related to the specified parameters can be evaluated using meticulous iterative simulations. The essential purpose of this study is to obtain the best performance in terms of proper impedance matching and broad impedance BW. Further, the simulation results of the surface current distribution at multiple resonances of the proposed antenna are also discussed in this section.

### 3.1. Influence of C_Sr1_ and C_Sr3_

**Figure 4:**
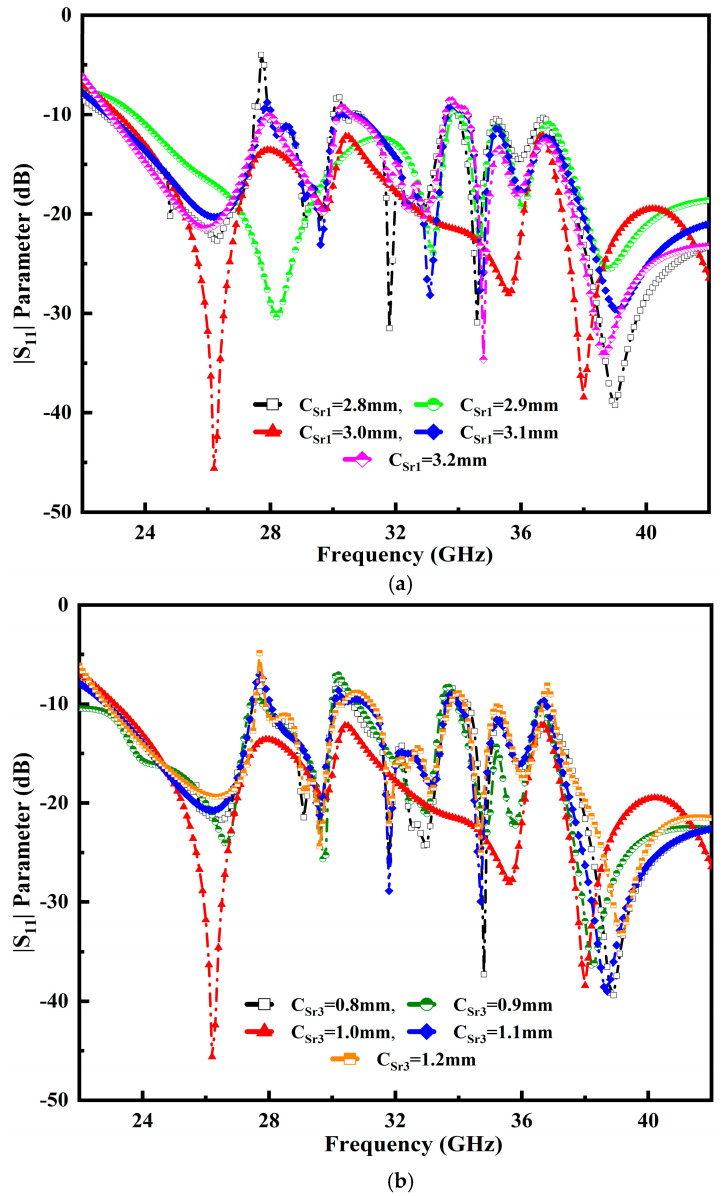
Influence of variable impacts: (**a**) a cavity radial stub 1 (C_Sr1_) and (**b**) cavity radial stub 3 (C_Sr3_) over the entire operable frequency range.

The composition of the semicircular radial cavity stub and elliptically loaded profiles is used to achieve antenna parameter accuracy. We embedded two cavity stubs from the initial design for the primary radiating purpose. The radiator of a developed antenna’s impedance-matching performance is influenced by the size of the cavity radial stub 1 (C_Sr1_) and cavity radial stub 3 (C_Sr3_). The proper impedance matching and broader BW are attained at a main radiator patch size of 3.0 mm, as illustrated in Figure 4a. Moreover, at the value of C_Sr3_ 1.0 mm, ideal matching is obtained as shown in Figure 4b.

### 3.2. Influence of S_C_W_C_ and W_FL_

**Figure 5:**
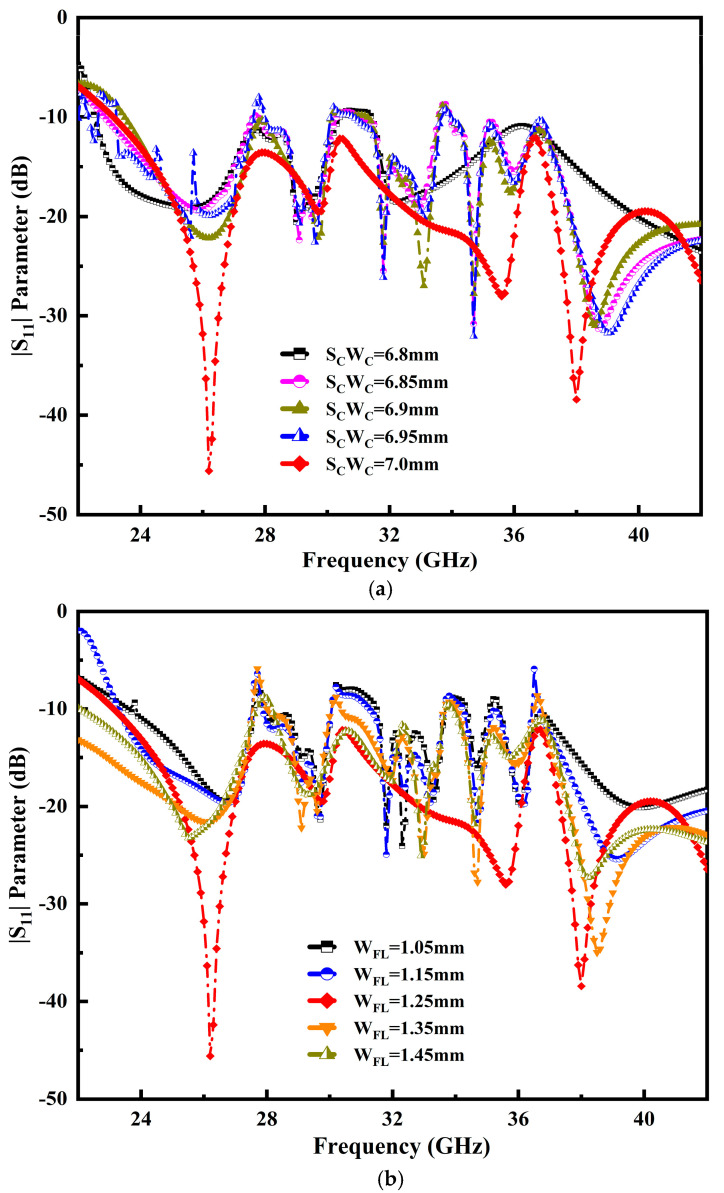
Influence of variation of variables: (**a**) return loss |S_11_| of the semicircular radiator (S_C_W_C_), (**b**) feedline width (W_FL_) over the operable frequency range.

Figure 5 portrays the semicircular cavity width (S_C_W_C_) and feedline width (W_FL_) that affect the overall performance of the proposed antenna. It is observed that the simulated result resonances obtained inclusive impedance BW performance. The S_C_W_C_ stub radiator values range from 6.8 mm to 7.0 mm. The value of 7.0 mm yields the best impedance-matching results, as illustrated in Figure 5a. The designed antenna feedline and two PDMGP grounds are the essential parts. An inverted triangle profile and a semicircular shaped radiator are coupled to ensure the proper transition. The feedline is used to excite the antenna radiator. In order to obtain the perfect impedance matching, selecting the appropriate dimensions of the feedline is crucial. The feedline width (W_FL_) fluctuation is between 1.05 mm to 1.45 mm and optimum results are obtained at the value of 1.25 mm, as depicted in Figure 5b. 

### 3.3. Influence of W_PDMGP_ and L_PDMGP_

**Figure 6:**
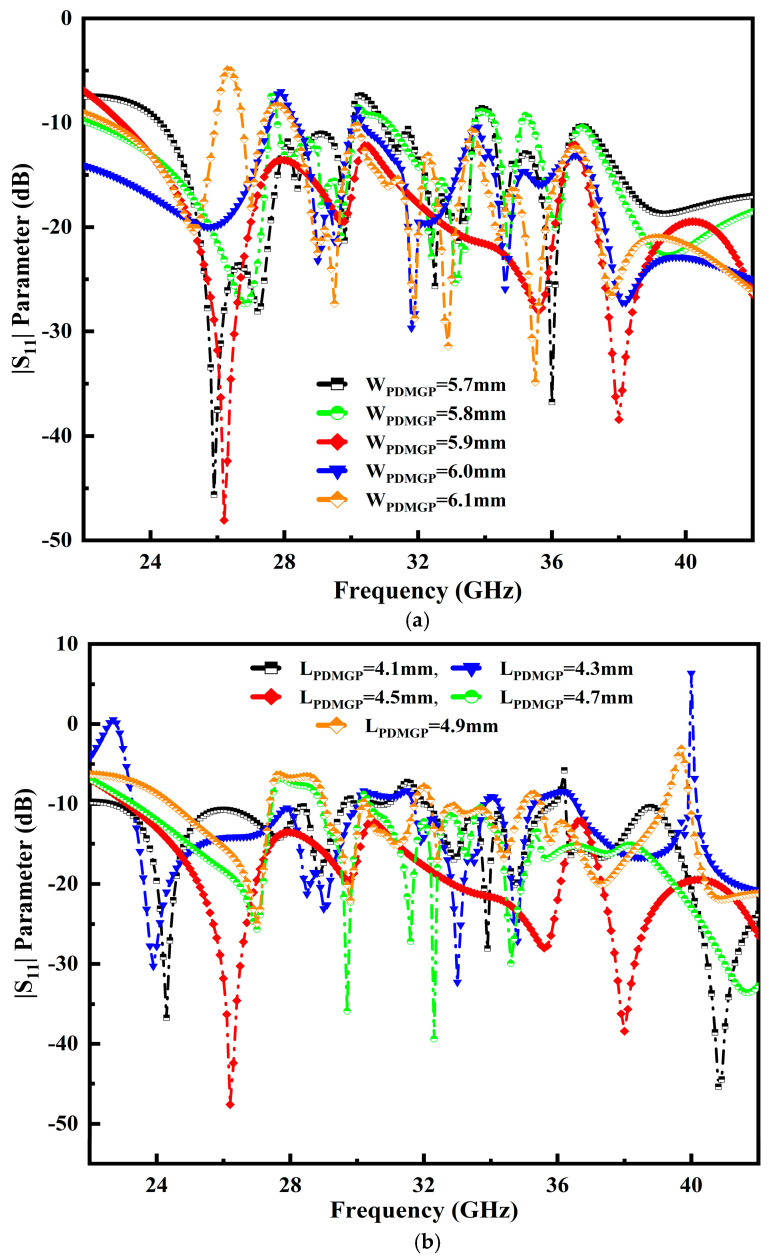
Performance of the parameters: (**a**) W_PDMGP_ and (**b**) L_PDMGP_.

The performance position of the CPW-e with defected ground surface is analyzed to improve the impedance characteristics. It is difficult to adjust the gap dimension between two partial defected ground planes with the center of the strip line. Figure 6a depicts the various opti-metric values used for the width of the partial defected metal ground planes (W_PDMGP_), ranging from 5.7 mm to 6.1 mm. It is observed that the proposed antenna attains proper matching at 5.9 mm, visible in a solid red line. Moreover, the effect of an L-shaped slot is truncated from the ground plane to obtain a broader BW. The variation in the length of partial defected metal ground plane (L_PDMGP_) values ranging from 4.1 mm to 4.9 mm is fixed. It is noted that the proposed antenna reaches proper matching at 4.5 mm, as portrayed in Figure 6b. 

### 3.4. Surface Current Distribution (J_SURF_)

**Figure 7:**
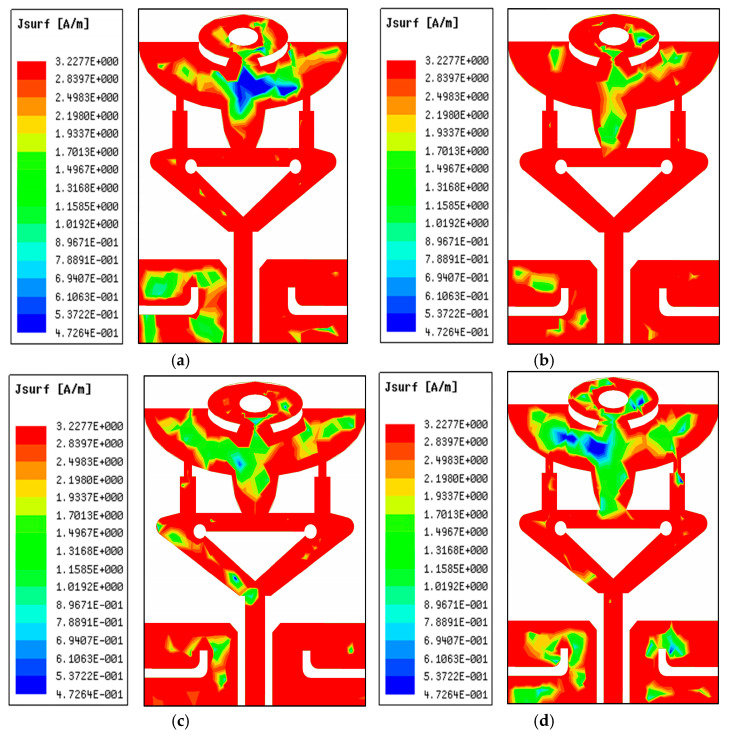
Surface current distribution (J_SURF_) at multiple frequencies: (**a**) 26.25 GHz, (**b**) 29.75 GHz, (**c**) 35.65 GHz, and (**d**) 38 GHz of the proposed antenna structure.

The current intensity across the radiator’s surface is examined and evaluated to validate the effectiveness. Figure 7a–d show the current distributed density over the proposed antenna lattice at various resonances. This is evident at 26.25 GHz; resonance shows that the surface current density is much stronger around the inner and outer edges of the semicircular inverted triangular-shaped patch, as shown in Figure 7a. An L-stub is loaded at PDMGP and transmission line with an inverted triangle at the higher current distribution at 29.75 GHz resonance, as illustrated in Figure 7b. Therefore, the resonant frequency is decreased and additional resonances are introduced with the second resonant of the antenna. This leads to the realizing of broadband resonance response features. It also indicates that the strong current is centered along the elliptical radiator’s structure of the 50 Ω CPW feedline. However, a slight fluctuation in current flow at higher resonances, such as 35.65 GHz and 38 GHz, can be seen on the radiator stubs and the slotted edge of the partially defected ground planes, as portrayed in Figure 7c,d.

## 4. Experimentally Validated Results

This section concentrates on the experimental outcomes of return loss |S_11_| (dB), peak realized gain (dBi), radiation efficiency (%) and far-field radiation patterns along the E–H plane. Further, the results are also investigated and analyzed in this section.

### 4.1. Return Loss |S_11_| Parameter

**Figure 8:**
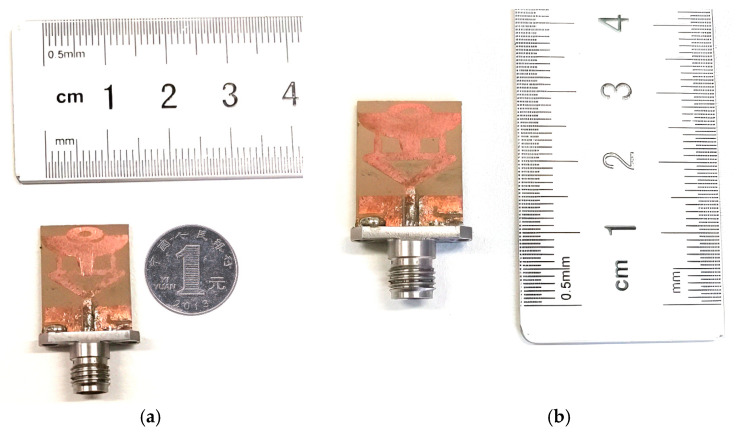
Fabricated antenna design model (**a**) antenna width, and (**b**) proposed antenna length.

**Figure 9:**
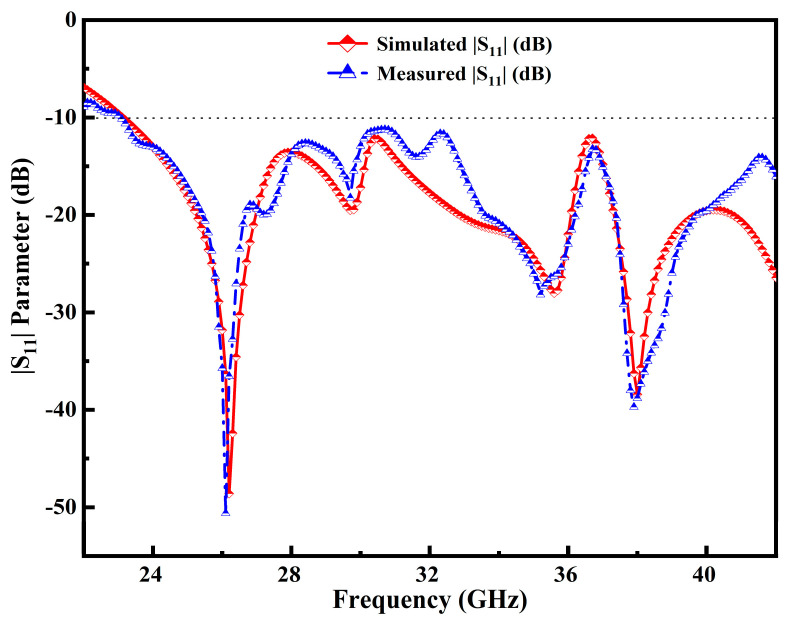
Simulation and measured results of |S_11_| parameter in the entire operable frequency range.

This segment presents the return loss |S_11_| performance of the manufactured and designed antenna. Figure 8a,b display a manufactured antenna sample. It can be seen that the middle pin of the SMA 50 Ω connector is soldered at the center of the CPW feedline and two more conductor pins are engraved with PDMGP. The |S_11_| performance for all of the experiment’s frequency sweeps is shown in Figure 8. The vector network analyzer (VNA) is precisely calibrated before assessing the fabricated antenna return loss |S_11_|. The microwave cable is connected to the port of the calibrated VNA N5244A Agilent technologies. The simulation and measurement results of the proposed design are depicted in Figure 9. Good agreement between the designed and fabricated models can be seen from Figure 9. However, there is a modest change in the resonances in the measurement’s outcome. 

The slight changes in the results may be due to the fabrication tolerances in the manufacturing of the substrate material, thickness, loss tangent or relative permittivity values, and improper soldering. It is clearly seen that the antenna’s simulation design has a broader impedance BW range from 23–42 GHz, resulting in 58.46%, which can be resonated at multiple frequencies. Moreover, the other two resonances are centered at 29.75 GHz and 35.65 GHz as depicted in Figure 9. As can be seen in the measured results (blue curve), at 35.65 GHz resonance there has been a slight shift in the resonance in the simulated result. Small variations have been obtained due to the glossy substrate material and the defective soldering of the SMA connector. Besides, higher resonance is observed at 38 GHz. The 28 GHz/38 GHz frequency band for mm-Wave 5G wireless communication systems may also be covered. 

### 4.2. Peak Realized Gain and Radiation Efficiency 

**Figure 10:**
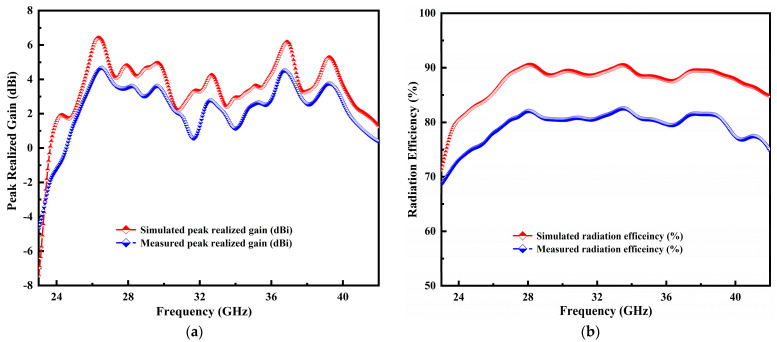
Simulation and measurement results of peak realized gain (**a**) and radiation efficiency (**b**) in the entire frequency span.

The simulation and measurement results of peak realized gain and radiation efficiency against the designated frequency span are presented in Figure 10a,b. The measured gain of the fabricated antenna is determined using the Friis transmission equation. The antenna model simulation result exhibited a high gain of 6.75 dBi and ~5.0 dBi at 27.2 GHz and 29.75 GHz. Similarly, an acceptable gain is observed at different frequency resonances. Values of 3.3 dBi and 3.05 dBi are achieved at 35.65 GHz and 38 GHz resonances. Moreover, two identical, known gain standard horn antennas were used to assess the peak realized gain of the manufactured antenna samples. During the measurement process, samples of the indoor antenna in the anechoic chamber were lost, resulting in a 0.5 dBi discrepancy in the peak realized gain, as illustrated in Figure 10a. 

Besides, the ratio of radiated to accepted power is known as the radiation efficiency, which is a crucial antenna characteristic. The simulated and measured radiation efficiency plot is shown in Figure 10b. The efficiency of the manufactured prototype is computed using an easy, reliable and rapid method based on the directivity/gain information. The measured radiation efficiency is used to obtain the following equation:

(1) $$\eta_{msd}=\frac{G_{msd}}{D_{smd}}$$

where $\eta_{msd}$ is denoted by the fraction of $G_{msd}$, and $D_{smd}$ which represents the designed antenna’s simulated and measured gain and directivity. The operable BW obtains the better simulated efficiency of 89% and tested 13.2% variance over the entire frequency span.

### 4.3. Radiation Pattern Performance

**Figure 11:**
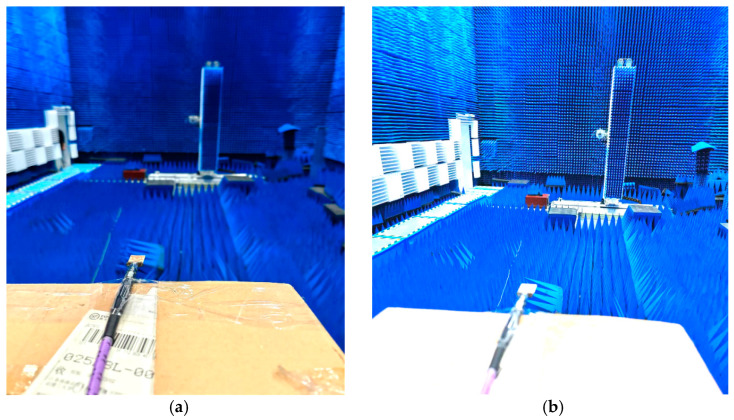
(**a**,**b**) Antenna under test (AUT) environment of anechoic chamber.

**Figure 12:**
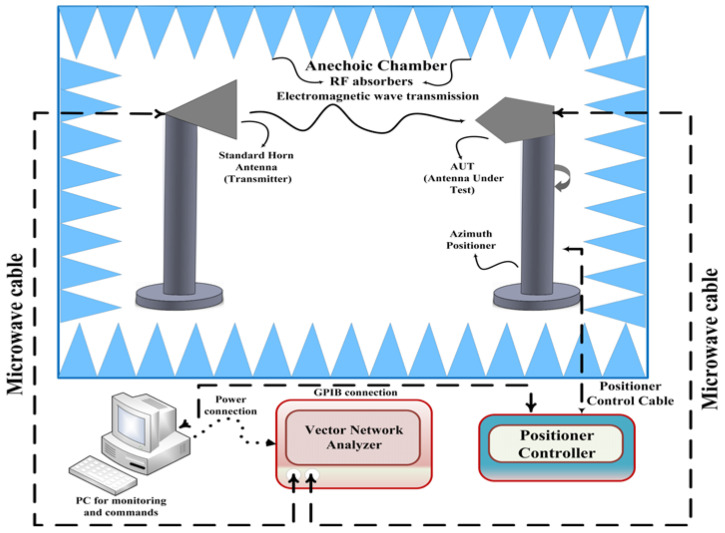
Visualization of the entire measurement setup.

The proposed antenna radiation patterns in the standard planes are measured inside the anechoic chamber under far-field conditions. In Figure 11a,b, the arrangement of the antenna under test (AUT) is depicted. The elevation (E-plane) and Azimuth (H-plane) of the manufactured antenna prototype are measured inside the anechoic chamber room. Moreover, the radio-absorbing material has been used to cover the indoor measurement facility walls. AUT was placed at the distance of the standard horn antenna. The positioner controller has been used to operate the proposed model table, which is wired via Ethernet to the computer. The AUT (receiver) and the typical standard horn antenna (transmitter) were set in a line of sight (L_O_S) at a specific distance. In addition, the AUT port is connected with a microwave cable (purple color) to the VNA as can be seen in Figure 11a. The installation of a computer allows for monitoring of measurement results, executing commands and storing measured data. The overall measurement setup is graphically represented in Figure 12.

**Figure 13:**
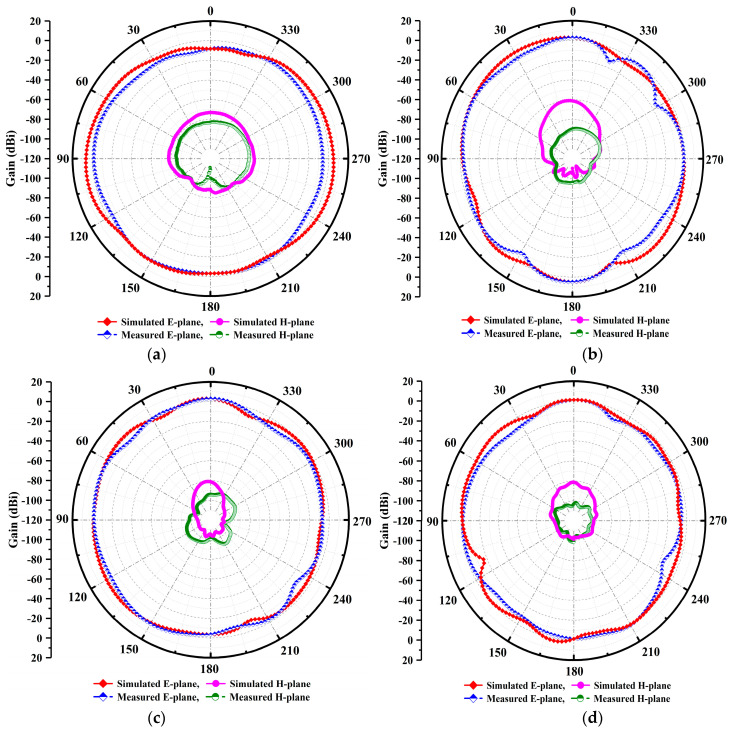
(**a**–**d**) Simulated and measured co-polarization and cross-polarization of E–H plane far-field patterns across the standard plane at multiple resonances.

Figure 13a–d display the radiation pattern performance of the designed and erected antenna model on the standard planes elevation, E-plane at ∅= 90° and azimuth H-plane at ∅=0°. The plots show that the proposed antenna model performs excellently and exhibits the intended resonant frequencies’ stable far field radiation pattern on standard planes. The displayed results can be observed consistently. The characteristics of the antenna radiation pattern are towards the $0^{{}^{\circ}}$ main lobe directions. The lower frequency resonance at 26.25 GHz of the radiation pattern is depicted in Figure 13a. It can be seen that the characteristics as essentially a fixed stable radiation pattern which radiates equally over $360^{{}^{\circ}}$. Moreover, the side lobes are visible at an angle of $300^{{}^{\circ}}$ and $340^{{}^{\circ}}$ in the measured results (blue curve) of the radiation pattern at 29.75 GHz as illustrated in Figure 13b. 

Furthermore, the antenna radiates $0^{{}^{\circ}}$ in the main lobe directions at 35.65 GHz, as portrayed in Figure 13c. However, there is a slight shift in the measured H-planes’ results as compared to the simulated results. Moreover, a perfect main lobe beam is seen at the 38 GHz resonance frequency in Figure 13d. The slotted antenna is primarily responsible for this observed variation in the radiation pattern at higher resonances. Because higher-order modes produce highly stable radiation characteristics in both standardized planes, the resultant far field fixed beam at higher frequencies’ radiation characteristics are visible. The aforementioned radiation patterns exhibit stability and act at specific resonances like fixed omnidirectional-shaped patterns. In addition, the patterns show a minor distortion at center frequencies. Due to chamber losses and glossy material, some asymmetry in the measured patterns is seen.

## 5. Comparison Analysis

The proposed design model of the antenna exhibits excellent performance results of broad impedance BW and peak realized gain with a low profile. A comparative analysis of the antenna key features with state-of-the-art work is shown in Table 2. The proposed antenna has a good BW performance of 58.46%, particularly for the 5G mm-Wave spectrum as compared with refs. [30,37,38,43,45]. The reported antennas achieved a good BW with a complicated assembly and complex geometrical structure. Another work-based CPW-fed broadband slotted antenna with different shapes has been investigated in [44]. The reported antenna structure obtained fair impedance BW at multiple frequencies with computational complexity. The results of the comparative analysis state that, compared to the proposed antenna, most of the reported works on 5G mm-Wave broadband antennas cover a limited frequency spectrum. It is concluded that the proposed model achieved a broad impedance BW of 58.46% as compared to the most examples in the reported work. 

## 6. Conclusions

A new semicircular inverted triangular shaped antenna based on a mixed-alternate approach has been presented for the 5G mm-Wave wireless communication system. The new design approach is based on the cavity, slots and loaded stubs. The proposed model exhibited broad impedance BW results at 58.46%, radiation efficiency of 89% and a high-gain of 6.75 dBi. The model is formed of an inverted-triangular arm radiator, metal ground plane and standard 50 Ω CPW feedline. The antenna elements were printed on low-cost Roger laminate material. The proposed antenna has a small dimension of 1.381λ_0_ × 1.08λ_0_ × 0.098λ_0_. The influence of multiple variables has been analyzed and discussed. The antenna model exhibited an excellent return loss performance, peak realized gain and stable far-field E–H radiation patterns. The intended antenna achieved in-band resonances with a broad impedance BW of 23–42 GHz. The suggested antenna demonstrated a good current distribution across the operable frequency range, a consistent omnidirectional radiation pattern and a reasonable peak realized gain performance of 6.75 dBi at lower frequency resonance of 26.25 GHz. The simulation and measured results are in close agreement and hence make the proposed antenna a competitive choice for 5G mm-Wave wireless communication applications. Moreover, the presented work may be further extended to design a MIMO antenna array topology.

## Figures and Tables

**Table 1:** Proposed antenna variables and optimized values (unit: mm).

Defined Variable	Optimized Values	Defined Variable	Optimized Values	Defined Variable	Optimized Values
S_C_W_C_	7	L_01_	0.8	W_PDMGP_	5.9
S_Cr_	0.5	L_02_	2	L_PDMGP_	4.5
C_Sr1_	3	L_03_	7.5	L_O_S_1_	3.5
C_Sr2_	1.2	L_04_ = L_05_	6	L_O_S_2_	1
C_Sr3_	1	G_AP1_	0.475	W_GP_SG_1_	1.9
C_Sr4_	0.5	G_AP2_	0.46	L_GP_SG_1_	1.5
C_Sr5_	1.5	r_01_	0.4	W_FL_	1.25
W_01_	0.5	F_T1_ = F_T2_	0.5	L_FL_	6
W_02_ = W_03_	1	C_F1_	0.5		
W_04_ = W_05_	1	W_O_S	0.5		

**Table 2:** Performance comparison analysis of the proposed antenna and recently reported works.

Ref./Year	Antenna Electrical Dimension (λ_0_) (L × W × H)	Substrate Material	Freq. Spect. Coverage (GHz)	Fract. Imp. BW (%)	Peak Gain (dBi)	Radiation Performance
Proposed work	1.38λ_0_ × 1.08λ_0_ × 0.098λ_0_	Roger RT/3010^TM^	23–42	58.46	6.75	Monopole Like (M.L)
[30]-2022	2.91λ_0_ × 1.30λ_0_ × 0.052λ_0_	Roger RO4003C	26–40	42.4	1.5–7.5	M.L
[35]-2020	3.475λ_0_ × 3.475λ_0_ × 0.07λ_0_	Roger RT/5880	27–29	7.15	12	M.L
[36]-2019	1.45λ_0_ × 1.80λ_0_ × 0.045λ_0_	Nelco NY9220	27–33	20	6–7.2	M.L
[37]-2022	1.13λ_0_ × 1.13λ_0_ × 0.022λ_0_	Roger RT/5880	25.98–35.38	30.64	4.86	M.L
[38]-2022	1.96λ_0_ × 2.09λ_0_ × 0.14λ_0_	Roger RT/5880	26–40	42.4	>7	M.L
[39]-2022	1.02λ_0_ × 1.02λ_0_ × 0.061λ_0_	Rogers 4350B	23.1–29.94	25.8	6.56	M.L
[40]-2022	1.76λ_0_ × 1.76λ_0_ × 0.07λ_0_	Rogers Ultralam 1250	26.4–31.6	17.93	8.0	M.L
[41]-2021	1.75λ_0_ × 1.75λ_0_ × 0.069λ_0_	Roger RT/5880	26.154–31	17	5.22	M.L
[43]-2021	1.67λ_0_ × 3.17λ_0_ × 0.042λ_0_	Arlon Di clad 880^TM^	25–35.5	34.73	11	M.L
[44]-2022	1.06λ_0_ × 0.92λ_0_ × 0.09λ_0_	FR4 Epoxy	26.43–28.47 32.65–36.15 39.57–44.63	7.43 10.17 12.01	4.93	M.L
[45]-2019	1.32λ_0_ × 1.32λ_0_ × 0.054λ_0_	Taconic TLY/Rogers 4450	31.5–50	45.4	8.5	M.L

## Data Availability

The data used to support the findings of this study are included within the article.
